# Impact of Thermal Processing on the Composition of Secondary Metabolites of Ginger Rhizome—A Review

**DOI:** 10.3390/foods11213484

**Published:** 2022-11-02

**Authors:** Justyna Zagórska, Lidia Czernicka-Boś, Wirginia Kukula-Koch, Radosław Szalak, Wojciech Koch

**Affiliations:** 1 Department of Food and Nutrition, Medical University of Lublin, 4a Chodzki Str., 20-093 Lublin, Poland; 2 Department of Pharmacognosy with Medical Plants Garden, Medical University of Lublin, 1 Chodzki Str., 20-093 Lublin, Poland; 3 Department of Animal Anatomy and Histology, Faculty of Veterinary Medicine, University of Life Sciences, 12 Akademicka Str., 20-950 Lublin, Poland

**Keywords:** *Zingiber officinale* Rosc., thermal treatment, transformation of components, dietary supplements, composition

## Abstract

Ginger (*Zingiber officinale* Rosc.) is both a commonly used spice, and an ingredient of various dietary supplements and medications. Its diverse applications result from the range of health benefits that this plant brings thanks to the presence of active compounds (secondary metabolites) in the matrix. Even if several studies underline a stronger pharmacological activity of fresh ginger rhizomes, the unprocessed plant is relatively rarely used. Ginger rhizomes are subjected to thermal processing, such as boiling, blanching, steam drying and others, at different temperature and time settings. Additionally, freeze-drying of the rhizomes is used as the first step in the preparation of raw material. It was proved that the composition of secondary metabolites of the *Zingiber officinale* rhizome changes upon the influence of temperature. Therefore, the aim of the review was to put together scientific results on the impact of traditional and unconventional methods of heat treatment on ginger rhizomes and to show the compositional differences that they induce in the plant matrix. Variations in the content and the transformation of some compounds into other metabolites will be also discussed, with particular attention paid to two major groups of secondary metabolites present in the plant, namely, phenolics and terpenes.

## 1. Introduction

Food of plant origin, including functional foods and dietary supplements, plays a significant role in the proper functioning of the human body. Plants produce a large variety of bioactive substances and phytochemicals that prevent the occurrence of chronic diseases that are based on inflammatory states and free-radical action, such as cancer, metabolic syndrome or aging. Vegetables, fruits, herbs and spices offer a more diverse and larger group of bioactives than drugs. Plants are certainly the source of chemicals that, after recognition of their pharmacological action, are often included in pharmaceuticals [1]. During the preparation of meals, most food products are thermally processed or pretreated to facilitate the digestive process in the digestive system and to enhance their taste and nutritional value. Food processing also aims to extend shelf life, facilitate transport, inactivate dangerous pathogens and give the appropriate texture. These processes usually involve heating, freezing or grinding of food products, which may result in the changes in the initial structure of a product or even in the destruction of bioactive compounds. Thermal treatments can be carried out in conventional ways that include cooking, blanching, freezing, and microwave heating, and in an unconventional way, that is, by using freeze-drying. Each of these processes differently influences the bioavailability of phytochemicals from the plant material.

**Figure 1:**
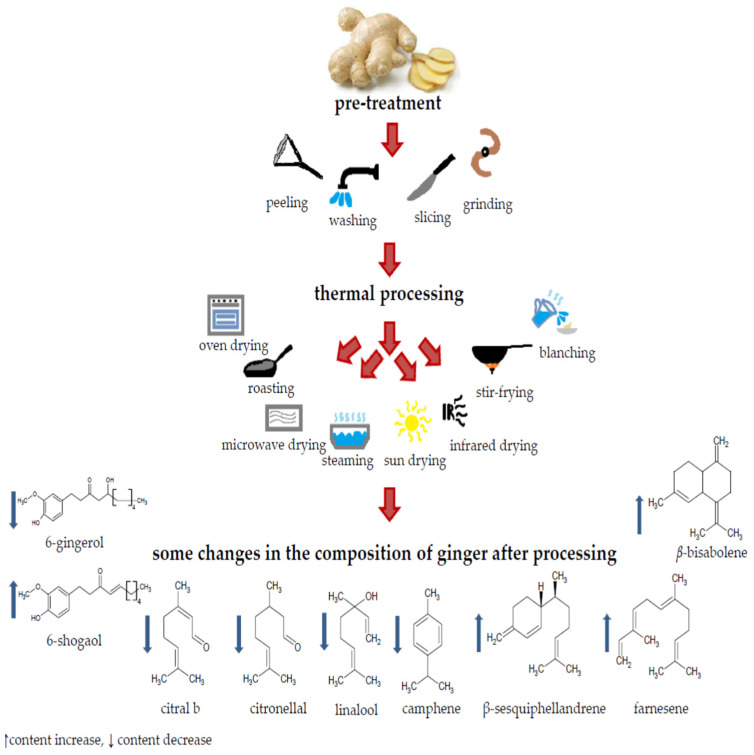
Schematic drawing of ginger processing.

Food processing procedures are seen as the main factors in the destruction and alteration of phytochemicals’ content, often reducing their activity and quantity. Based on these observations, it is of the highest importance to adjust the conditions of thermal processing in a way to retain the sensory, nutritional and pharmacological properties, and at the same time, prolong the shelf life of a product to support successful transportation (see Figure 1) [2,3,4,5,6].

### 1.1. Ginger (Zingiber officinale Rosc.)

Ginger (*Zingiber officinale* Rosc.) belongs to the Zingiberaceae family, which includes over 1300 species and is found in Southeast Asia and India. Ginger rhizome is a popular aromatic spice used around the world in cuisine as an addition to cakes, drinks, meats and sushi due to its characteristic sharp taste [7,8]. The first references to the medical properties of ginger can be found in works dating back over 2000 years that describe the traditional use of ginger rhizome in the treatment of colds, vomiting, nausea, stomach upset and cough [9,10]. Because of its wide biological activity, ginger and its products are widely used in the food industry as a spice, as a flavoring agent in foods and beverages, and in the production of dietary supplements and functional foods [11]. The main pharmacological properties of ginger result from the presence of phenolic and terpene compounds. In addition to them, the rhizome contains carbohydrates, lipids, glycosides, alkaloids, saponins, steroids, tannins and elements (K, Mg, Mn, Zn, Fe) [12]. The group of phenolic compounds includes gingerols; their dehydration products, shogaols; and their derivatives. The main components of this group are 6-shogaol and 6-gingerol, which are present together with their derivatives: paradols, zingeron and diarylheptanoids [13]. Apart from the hydrophilic fraction, the ginger rhizome contains essential oil (1–3%), which includes terpene compounds such as monoterpenes: 1,8-cineole, linalool, borneol, neral, geraniol, camphene, limonene, myrcene, α-, and *β*-phellandrene, α-and *β*-pinene, citronellal, geranial, citral, and terpineol; and sesquiterpenes: zingiberene, ar-turmerone, farnesene, *β*-bisabolene, *β*-sesquiphellandrene, zingiberol, zingiberenol and α-turmerone [14,15]. The chemical composition of ginger is rich and complicated, and varies in its qualitative and quantitative content depending on the place of origin, method of processing/preparation, and whether the raw material was fresh or dried [16].

The available literature data indicate a number of pharmacological activities of ginger, including: antioxidant [17], antibacterial [18,19,20], antiparasitic [21,22], anti-inflammatory [13,23], antiaggregation [9], and analgesic [24]. Numerous studies have shown that ginger and its ingredients can prevent cardiovascular disease and related risk factors: obesity [25,26] and diabetes [27]. The effects of ginger have been documented as alleviating gastrointestinal [10,28] and respiratory [29] dysfunctions; inhibiting chemotherapy-induced nausea and vomiting [30] and nausea in pregnancy [31]; and showing anti-inflammatory properties that are used in the treatment of arthritis [24]. Research into the chemopreventive properties of ginger extracts against various types of cancer is also interesting. The mechanism of ginger’s antitumor activity is to regulate mediators involved in key cellular processes including apoptosis, cell-cycle arrest, and the inhibition of cell proliferation, cancer cell proliferation and angiogenesis [32,33,34]. Over the past several decades, an increased interest in studying the pharmacological effects of ginger and its components on the central nervous system was noted. The obtained scientific results proved that ginger could be used in the treatment of neurodegenerative diseases such as Alzheimer’s, Parkinson’s, senile dementia and depression. The neuroprotective effect of ginger is due to its ability to improve cognitive functions and behavioral disturbances, and due to its strong antioxidant properties that inhibit the lipid peroxidation of nerve cell membranes [7,8,14,35].

### 1.2. Application in Food

Ginger rhizome is widely used in medicine, but also as an ingredient in cosmetics, dietary supplements, functional foods and as a popular seasoning in the kitchen due to its characteristic smell and taste [12]. In ginger, we distinguish fractions of volatile compounds that constitute the essential oil (terpenes), which is responsible for the fresh smell of the raw material, and nonvolatile fractions, which give it a pungent taste (phenolic compounds) [7]. The searing, pungent taste of ginger can irritate the digestive system, which in turn, contributes to reduced ginger consumption. In order to reduce the harsh taste of ginger, various methods of processing the raw material have been developed, which were simultaneously tested for their impact on the content of active compounds, on the shelf life of the plant and on its taste [16]. The methods of ginger rhizome processing include drying, cooking, baking, blanching, microwave heating, candying and the fermentation process in which marinated ginger is obtained. An interesting process used for the preparation of ginger rhizomes is the freeze-drying method, as it can be carried out both in industrial and laboratory conditions and it provides mild conditions for the initial preparation of this food product [5,36]. The available data indicated that the introduction of ginger into food products was first carried out with beer and gingerbread cookies [15]. These days, marinated rhizome is a popular addition to sushi, and candied rhizomes are often used for baking. Boiled, fried or baked ginger is a tasty and useful ingredient in meat dishes as well. In these products, ginger is known to enhance the taste of meals, but also to extend their shelf-life due to its antioxidant and antibacterial properties [4,18]. It is worth noting that ginger, along with its preparations, is one of the most commonly used natural ingredients applied in dietary supplements to alleviate vomiting and nausea (especially during pregnancy) or knee arthritis [11,37,38].

## 2. Materials and Methods

A comprehensive search for scientific articles on the reviewed topic was carried out using databases such as PubMed, Scoputs, Science Direct and Web of Science. When searching for information, the following keywords were used: “*Zingiber officinale*”, “ginger”, “thermal treatment”, “drying”, “microwave”, “cook”, “roast”, “boil”, “fry”, “steam”, “ blanch”, “lyophilization”, “composition”, “ingredients”, and “activity”. This study includes only those articles in which the data from the obtained research were related to the raw ginger rhizome, and the references to these studies were also checked. The search was limited to publications in English. Based on the criteria and keywords listed, 42 articles were selected for their findings on the effects of processing and thermal treatments on the activity and chemical composition of ginger rhizomes. The review included works published in the years 1989–2022.

## 3. Food Processing of Ginger Rhizomes

Food processing is mainly based on heat treatment—that is, increasing the temperature by using hot air, hot water, electromagnetic waves or oil. Additionally, some types of processing may include storing food in refrigerators or freezers. All processes affect the bioavailability and bioaccessibility of active compounds from the plant matrix in a different way. Physical factors such as temperature and biological factors, i.e., enzymatic activity, can cause a loss of components present in the plant matrix. Even if producers and consumers are concerned about the quality of the food product, some processes may cause the degradation of phytochemicals, analogically reducing their amount in food and reducing the bioactivity of the food product [4]. On the other hand, processing may also lead to some positive chemical or physical modifications, allowing for the better absorption or release of active compounds from food in the digestive system [5]. Therefore, this review pus together both these aspects of processing in relation to broadly consumed ginger rhizomes.

### 3.1. Cooking/Thermal Processing

Cooking is a common method of preparing food that can be conducted in two ways. The first way is to put the product into the boiling water. The second one can be achieved by putting the product into cold water, and then bringing the water to the boiling point. During cooking, the interior plant tissue is heated by the convection of hot water. As it was proved, cooking, similar to blanching, is a culinary technique in which a large volume of water is used. As a result of thermal treatment, changes in the functioning and structure of the cell walls, membranes and cell organelles are induced. Most often, these changes result in a significant loss of nutrients that pass from the processed product to the water environment. The degree of the loss depends on the amount of water used in the processing and the duration time of boiling (see Table 1) [6].

### 3.2. Roasting

Heat treatment methods also include roasting, which uses hot, dry air that interacts with a food product. In their studies, Schaller and Schieberle (2020) [39,40] analyzed the effect of ginger roasting on the composition of its essential oil. Twenty aromatic compounds were identified in fresh ginger oil, whereas in roasted ginger oil, twenty-two were identified. Acetaldehyde was present only in fresh ginger oil, whereas 3-hydroxy-4,5-dimethyl-2(5H)-furanone, 4-hydroxy-2,5-dimethyl-3(2H)-furanone and 3-(methylthio)-propanal were present only in roasted ginger oil. As a result of thermal treatment, the content of individual components of the oil also changed. Comparing the amounts of selected ingredients in fresh ginger oil and in oil from roasted ginger, it was noticed that the content of (S)-linalool decreased from 1700 in the fresh rhizome to 920 µg/kg in the processed one, and the content of (E)-2-octenal from 1800 to 930 µg/kg. Additionally, in fresh ginger oil, the content of (S)-citronellal was calculated as 89 µg/kg, geraniol as 2100 µg/kg, 3-hydroxy-4,5-dimethyl-2(5H)-furanone as <0.1 µg/kg, 4-hydroxy-2,5-dimethyl 3 (2H)-furanone as <0.1 µg/kg, vanillin as 350 µg/kg, 3-(methylthio)-propanal as <0.1 µg/kg, neral as 25,000 µg/kg and (E)-2-octanal as 1470 µg/kg; after thermal treatment, the content of the listed components in the oil increased to 310, 8400, 1.5, 780, 990, 80, 47,000 and 3000 µg/kg, respectively (Table 1).

### 3.3. Blanching

Blanching involves immersing the product in the boiling water for several seconds and then cooling it in cold water. It is considered as a pretreatment and is usually used to inactivate certain enzymes in vegetables and fruits before freezing, or as a process to remove larvae and bacteria from leafy vegetables to keep them crispy and fresh [6]. The scientific articles on ginger rhizome blanching refer to the study of the content of various compounds.

In the work of Kumar et al. (2018) [41], it was noted that in the production of ginger candies, which involved blanching ginger rhizomes for 10 min, the total phenolic content (TPC) was 121.83 mg/100 g, in comparison to unblanched ginger where the concentration was 118.01 mg/100 g. It occurred that blanching slightly increased the TPC of ginger rhizomes (Table 1). Other studies revealed that blanching for 10 min at 100 °C in both water and steam increased the content of essential oil in the rhizome to 1.38% DW (dry weight) and 1.25% DW in comparison to 1.20% DW in the fresh rhizome. This study also assessed the effect of heating the ground rhizome on the oil content. As a result, the measured amount of essential oil was the highest in blanched rhizomes, followed by heated and fresh rhizomes. Processing increased the quantity of the recovered essential oil. As during blanching, the ginger was not ground initially; thus, the volatile compounds were protected from evaporation. It is worth emphasizing that the partial loss of essential oils may have occurred during lyophilization, in which a vacuum is applied [42]. However, in the article of Gan et al. (2016) [43], blanching sliced ginger in a water bath at 70 °C resulted in a decrease in the content of 6-gingerol. In unblanched ginger, the content of the tested compound was at the level of 1.273%. With the extension of the blanching time, the concentration of 6-gingerol decreased. After 5 min of blanching it was 0.773%, after 15 min 0.348%, and after 30 min it decreased more than 4 times compared to unblanched ginger and was equal to 0.294%.

### 3.4. Steam Cooking and Steam Heating

Steaming is one of the cooking techniques that uses steam from boiling water without immersing the food. There are only a few articles available describing the use of this technique in the processing of ginger rhizome. According to Nam et al. (2020) [44], steaming increased the content of 1-dehydro-6-gingerdione in the extract from ginger rhizome by 375% compared to the extract obtained from unprocessed ginger. The content of this compound in the crude extract was 0.04%, and in the steamed ginger extract 0.19% (Table 1). One study compared the content of 6-gingerol, 6-shogaol, and the total soluble solid yield (TSSY) in fresh and steamed ginger extracts; in the first extract, the values of these parameters were 2.76, 1.42 and 32.96 mg/g, respectively, whereas in the case of steamed ginger extract, the corresponding values were as follows: 1.51, 2.02 and 22.83 mg/g. Comparing the described data, it was noticed that in the steamed ginger extract, the total soluble solid yield (TSSY) and 6-gingerol decreased, whereas the 6-shogaol content increased [45]. Shogaols are considered products derived from the dehydration of gingerols. These changes occur during long-term storage or as a result of thermal treatment, e.g., heating or drying [46,47,48]. This might explain the simultaneous decrease in the concentration of 6-gingerol and the increase in the 6-shogaol content in the steamed ginger extract.

When analyzing the composition of the essential oil obtained from fresh, steamed and dried ginger, it was hard to see any dependence between thermal treatment and quantitative composition. The content of some substances was higher in fresh ginger oil, such as zingiberene, citral, geraniol, camphene, sabinene and 1,8-cineole. In turn, oil from processed ginger contained higher amounts of ar-curcumene, *β*-bisabolene, farnesene, *β*-sesquiphellandrene and geranyl acetate than fresh ginger oil. The main ingredients of fresh ginger oil are citral (24.9%) and zingiberene (17.1%), whereas the oil from the processed plant contains the most *β*-bisabolene (18.7%) and ar-curcumene (18.1%) [49]. An increase in ar-curcumene may be due to the thermal conversion of zingiberene during steam-heated processing of ginger rhizomes [50].

The differences in the composition of fresh and steamed ginger ethyl acetate extract were investigated using the GC method by Takahashi et al. (2011) [49]. In both extracts, the compounds with the highest content were zingiberene, bisabolene and *β*-sesquiphellandrene; after thermal treatment, the content of each increased, from 26.2 to 30.4%, 10.8 to 20.2% and 10.3 to 14.8%, respectively. The concentration of ar-curcumene, farnesene and geranyl acetate in the steam-heated ginger extract was also elevated. Additionally, 6-shogaol was detected only in the processed and not in the fresh ginger extract. On the other hand, as a result of the thermal treatment, the content of 1,8-cineole, camphene, sabinene and 6-gingerol decreased. In addition, citral that was found in the fresh ginger extract was finally not detected in the processed ginger extract. Another phenolic component, 6-shogaol, was found only in the processed ginger extract at the level of 25.6%. As for gingerols, their content in the fresh ginger extract was as follows: 6-gingerol—65.2%, 8-gingerol—6.4%, 10-gingerol—10.9%, whereas the content of these compounds in the extract from the steam heated ginger was: 46.1%, 7.1% and 8.6%, respectively. Analyzing these data, it can be seen that when the extract was under the influence of the treatment, the content of 6- and 10-gingerol decreased, whereas the content of 8-gingerol slightly increased.

### 3.5. Stir-Frying

Stir-frying is a method of thermal processing food that originates from Asia. It has recently been gaining popularity in other regions of the world as well. In general, it is based on frying in a pan for a short time while intensively mixing the product. In this culinary technique, food is heated by transferring heat from the pan to the product using a small amount of oil [51]. According to Li et al. (2016) [52], fresh and stir-fried ginger contained 8- and 10-shogaol, 6- and 8-paradol, 3- or 5-acetoxy-6-gingerdiol, 5-hydroxy-1-(4-dihydroxy-3-methoxyphenyl)-7-(3,4-dihydroxyphenyl)-3-heptanone,methyl-6-gingerol and 6-gingerdiol. In turn, 8-gingerdione was present only in the fresh ginger extract. The content of the tested compounds was as follows: 6-shogaol, 0.067 ± 0.005 (fresh ginger extract) and 5.843 ± 0.011 mg/g (stir-frying ginger extract); 6-gingerol, 2.035 ± 0.023 and 5.561 ± 0.012 mg/g; 8-gingerol, 0.128 ± 0.010 and 1.480 ± 0.019 mg/g; and 10-gingerol, 0.058 ± 0.002 and 1.425 ± 0.012 mg/g. In the case of zingerone, its content in the extract from the unprocessed plant was below the limit of quantification, whereas in the stir-frying ginger extract it was 0.691 ± 0.004 mg/g. The TPC parameter increased in the stir-fried ginger extract to 22.24 mg GAE/g (gallic acid equivalents) from 8.46 mg GAE/g. Obtained data revealed that the thermal treatment of ginger had a positive effect on the composition of its extract, contributing to the appearance of several new compounds. Moreover, it increased the content of five main components of ginger, which were analyzed quantitatively in this study (Table 1).

### 3.6. Drying

Drying in the sun and drying with hot air are the methods of thermal treatment that belong to the conventional methods of heating. In a classical way, they involve the transport of heat from its source to the product. Heating is mostly due to the conduction and convection of hot air; therefore, these are relatively time-consuming methods [53].

Apart from them, there are methods that use vacuum conditions or microwaves in order to shorten the drying process.

#### 3.6.1. Air-Drying

Essential oil obtained from ginger dried in the sun was characterized by a lower content of the determined components compared to the oil from fresh rhizomes. The exception was citral, the content of which increased in the oil from a dried plant (24.9% in fresh and 70.5% in dried) (Table 1) [49]. The effect of drying on the content of polyphenolic compounds was also investigated. The sum of the phenolic compounds ((+)-catechin, (−)-epicatechin, rutin, myricetin, trans-resveratrol, quercetin, naringenin and kaempferol) increased in the extracts of both 9- and 12-month-old sun-dried ginger rhizomes. The determined sum of all compounds in the fresh ginger extract was 38.42 (9-month-old ginger rhizomes at 9 months after planting) and 55.32 mg/100 g in 12-month-old rhizomes; after drying, this content increased to 415.91 and 423.65 mg/100 g, respectively. The TPC also increased from 21.84 ± 3.21 to 163.84 ± 1.18 mg GAE/g and from 39.81 ± 2.25 to 131.86 ± 2.80 mg GAE/g for the processed 9- and 12-month-old rhizomes, respectively [54].

In other studies, Gümüşay et al. (2015) [55] evaluated the impact of sun-drying ginger on the content of glutathione (GSH), cysteine (Cys) and TPC in the extract. They revealed that the content of GSH decreased by 99.97%, Cys by 94.82% and TPC by 76.34% compared to the content in the extract from fresh ginger. A significant reduction in the amount of glutathione and cysteine in the extract could result from the degradation of these compounds during thermal treatment, i.e., as a result of the oxygen and heat impact. The conducted study also revealed a detrimental influence of heat on the content of ascorbic acid (AA) in ginger rhizome. In the unprocessed ginger extract, the concentration of AA was 79.86 ± 4.37 mg/100 g DW, whereas in the sun-dried ginger extract this vitamin was not detected.

#### 3.6.2. Drying

An interesting correlation was noticed in the studies of Gan and co-investigators [43]. According to the authors, the drying of ginger rhizomes under varying temperature conditions and in variable relative humidity conditions had a positive effect on the quantity of 6-gingerol compared to the samples dried under constant conditions of these parameters. The most advantageous conditions for keeping the content of 6-gingerol as high as possible include drying at variable temperatures from 30 °C to 40 °C and at a relative humidity dropping from 30% to 10%. The content of this phenolic compound in ginger subjected to varying conditions was equal to 1.623%. On the other hand, ginger dried at a relative humidity of 15% and at a temperature of 60 °C was characterized by the lowest concentration of 6-gingerol (1.047%) among all dried ginger samples (Table 1).

In another study, the content of 6-, 8- and 10-gingerol and 6-shogaol in commercially dried ginger and fresh white and yellow ginger were determined. The content of all gingerols in the fresh ginger extracts, of both the white and yellow varieties, was much higher than in dried ginger. The content of 6-gingerol, 8-gingerol and 10-gingerol was highest in fresh yellow ginger, then in fresh white ginger, and the lowest was in dried ginger. On the other hand, the content of 6-shogaol in the dried ginger extract was higher than in the samples from fresh yellow and white ginger, where it was at a similar level. The content of compounds in the extract was as follows: 6-gingerol—11.38%, 27.56% and 33.96%; 8-gingerol—2.17%, 3.20% and 4.64%; 10-gingerol—3.44%, 5.38% and 7.91%; and 6-shogaol—3.29%, 0.36% and 0.35%, in the extracts from dried ginger, and fresh white and yellow ginger, respectively. Interestingly, the concentration of 6-gingerol was almost 3 times higher in fresh yellow ginger than in dried ginger, whereas the content of 8- and 10-gingerol was more than twice as high. On the other hand, the amount of 6-shogaol was over 9 times higher in dried ginger than in fresh ginger, both in the yellow and white varieties. The decrease in gingerol content and the increase in 6-shogaol in dried ginger extract may result from the dehydration of the first compounds in the process of commercial drying and their transformation into corresponding shogaols [56].

Li et al. (2016) [52] identified compounds in dried ginger extract that were not present in fresh ginger extract. Compounds resulting from drying were 5-hydroxy-1-(4-dihydroxy-3-methoxyphenyl)-7-(3,4-dihydroxyphenyl)-3-heptanone, 6-gingerdiol, methyl-6-gingerol, 3- or 5-acetoxy-6-gingerdiol, 6-paradol, 8-shogaol, 8-paradol and 10-shogaol. In contrast, the compound that was present only in the fresh ginger extract was 8-gingerdione. The remaining components were quantified as: 6-gingerol, 2.035 ± 0.023 mg/g; 8-gingerol, 0.128 ± 0.010 mg/g; 10-gingerol, 0.058 ± 0.002 mg/g; and 6-shogaol, 0.067 ± 0.005 mg/g, whereas the amount of zingerone was below the limit of quantification. After drying, their content increased to: 8.715 ± 0.029, 2.357 ± 0.008, 2.263 ± 0.014, 3.680 ± 0.030 and 0.158 ± 0.007 mg/g for the particular compounds, respectively. After the treatment, the TPC value also increased from 8.46 to 27.40 mg GAE/g.

#### 3.6.3. Hot-Air-Drying, Convection Drying, Oven Drying, and Vacuum-Drying Oven

Depending on the drying technique and conditions, the researchers obtained different results. Drying ginger with hot air in an oven affected the composition of its essential oil. Forty-nine compounds were detected in fresh ginger oil, and fifty compounds in oven-dried oil. The content of individual components increased or decreased as a result of drying. The content of some of the main ingredients of ginger essential oil, such as zingiberene, geranial, *β*-sesquiphellandrene, α-curcumene and *β*-bisabolene, varied from 22.76, 14.50, 7.01, 2.78 and 3.25% in fresh rhizome extract to 38.43, 9.32, 9.93, 5.31 and 5.24% for dried plants, respectively. Moreover, it was revealed that this drying method resulted in the formation of various esters, such as bornyl acetate and propanoic acid (Table 1) [57]. The long-term exposure of ginger to oxygen could contribute to the synthesis of these compounds as a result of the esterification of alcohols to esters [58]. As a result of drying, the gingerol content in the extract was reduced, whereas the 6-shogaol concentration increased. The fresh ginger extract contained 5.91 mg/g of 6-gingerol, 2.52 mg/g of 8-gingerol, 2.62 mg/g of 10-gingerol and 0.09 mg/g of 6-shogaol (per DW). However, in the oven-dried ginger extract, the content of these compounds was at the level of 2.50, 2.15, 2.33 and 0.214 mg/g. The total content of phenols (TPC) in the extract after treatment decreased from 11.97 ± 0.33 to 9.69 ± 0.54 mg GAE/g DW, and the total content of flavonoids (TFC) from 13.49 ± 0.36 to 12.08 ± 1.17 mg rutin/g DW [57].

In another study of fresh ginger oil, 54 components were identified using gas chromatography-mass spectrometry (GC-MS); the number of identified constituents of oil increased from 54 components to 59 (at 50 °C) and 61 (at 60 and 70 °C) in hot-air-dried ginger oil. As a result of the thermal treatment, there were also changes in the content of the main volatile components. The content of 2,6-octadienal, 3,7-dimethyl-,(Z) and 2,6-octadienal, 3,7-dimethyl- was lower in each processed ginger oil compared to the fresh ginger oil. In the fresh ginger oil, the amount of the first compound was 5.56% and the second was 15.71%, whereas after drying it was 2.88 and 12.78% (at 50 °C), 3.12 and 13.39% (at 60 °C), and 2.20 and 11.00% (at 70 °C), respectively. Additionally, the content of benzene,1-(1,5-dimethyl-4-hexenyl)-4-methyl-; 1,3-cyclohexadiene,5-(1,5-dimethyl-4-hexenyl)-2-methyl-[S-(R*,S*)]; α-farnesene; and cyclohexene,3-(1,5-dimethyl-4-hexenyl)-6-methylene-,[S-(R*,S*)] increased as a result of drying regardless of the temperature used. In fresh ginger oil, dried at 50, 60 and 70 °C, the content of individual compounds was as follows: benzene,1-(1,5-dimethyl-4-hexenyl)-4-methyl—2.28, 4.09, 5.55 and 4.27%; 1,3-cyclohexadiene,5-(1,5-dimethyl-4-hexenyl)-2-methyl-[S-(R*,S *)]—28.12, 43.24, 41.96 and 45.97%; α-farnesene—6.90, 10.03, 10.95 and 11.11%; and cyclohexene,3-(1,5-dimethyl-4-hexenyl)-6-methylene-,[S-(R*,S*)]—7.64, 10.64, 9.77 and 11.54% [58].

The effect of convection drying was also investigated, as a result of which the content of essential oil in ginger rhizomes decreased to 2.9% *v*/*w*. For comparison, the amount of oil in fresh rhizomes was 3.2% *v*/*w*. The drying also reduced the sesquiterpene hydrocarbon content from 1963 µL/100 g DW to 1402 µL/100 g DW. On the other hand, the content of other compounds forming various chemical classes increased: monoterpene hydrocarbons from 619 to 791 µL/100 g DW, oxygenated monoterpenes from 405 to 445 µL/100 g, oxygenated sesquiterpenes from 146 to 158 µL/100 g, other aliphatic compounds from 6 to 7 µL/100 g and other oxygenated aliphatic compounds from 37 to 45 µL/100 g DW. The higher content of monoterpenes and the lower content of sesquiterpenes in dried ginger oil may have been due to the long-term exposure of ginger to oxygen during drying and the consequent degradation of the sesquiterpenes to monoterpenes [59].

Interesting results were presented by Chumroenphat et al. (2011) [54] concerning the influence of the drying temperature on the content of polyphenolic compounds. The effect of oven drying ginger rhizomes at various temperatures (40–70 °C) on the content of the sum of phenolic compounds ((+)-catechin + (−)-epicatechin + rutin + myricetin + trans-resveratrol + quercetin + naringenin + kaempferol) was investigated in extracts obtained from it. It was noted that in the case of 9-month-old rhizomes, the highest content was found in extracts from rhizomes dried at 60 °C, 441.85 mg/100 g, compared to extracts from fresh rhizomes, 38.42 mg/100 g. On the other hand, in the case of 12-month-old flocks, the content in extracts from fresh rhizomes was 55.32 mg/100 g, and the highest was found after drying in an oven at 70 °C and was 450.16 mg/100 g. The TPC value was the highest for rhizomes dried at 60 °C. For 9-month-old rhizomes it was 204.30 ± 2.27, and for 12-month-old rhizomes it was 219.31 ± 3.04 mg GAE/g, which was much higher than in fresh rhizome extracts, where it was 21.84 ± 3.21 and 39.81 ± 2.25 mg GAE/g, respectively.

In the work of Gümüşay et al. (2015) [55], as a result of drying in the oven, the content of the determined compounds decreased compared to their content in the fresh ginger extract. The loss of the components was as follows: glutathione, 99.12%; cysteine, 94.70%; and TPC, 73.80%. In addition, no ascorbic acid was detected in the oven-dried ginger extract, but was present in the fresh ginger extract (79.86 ± 4.37 mg/100 g DW).

Most of the research on the heat treatment of ginger rhizomes concerns its effect on the content of essential oil and phenolic compounds, but there are studies on the content of other ingredients. The work of Salem et al. shows that drying ginger in the oven also affected the amino acid content, which was doubled, and the starch content that was reduced by about 10 mg/g DW. Additionally, the protein content was more than doubled. Furthermore, drying resulted in a significant decrease in the number of lipids from different classes. From a nutritional point of view, fresh ginger appears to be a better choice than dried ginger due to its higher content in fatty acids and essential amino acids. As a result of the treatment, the content of glutathione, riboflavin and xylose also decreased. On the other hand, drying contributed to increased citric acid, valine and arginine levels in the extract. Both extracts differed in the content of gingerols and gingerol-related metabolites. The fresh ginger extract was characterized by a higher content of, e.g., 6-, 8- and 10-gingerol, whereas the dried ginger extract had 6-shogaol, phenylalanine and cinnamic acid at higher concentrations [60]. It is worth remembering that the drying process allows us to remove excess moisture from the plant. This contributes to the inhibition of spoilage, facilitates storage and extends the shelf life of the raw material. This, in turn, translates into lower economic losses for producers [61].

Huang et al. (2012) [62] also investigated the effect of drying on the content of ginger essential oil and found that changes in its composition occurred during oven drying. In total, 42 volatile compounds were identified in fresh ginger essential oil, but after drying, this number increased to 45. The content of *β*-phellandrene, zingiberene, *β*-bisabolene and *β*-sesquiphellandrene in fresh ginger oil was 12.9%, 27.8%, 5.7% and 10.4%, respectively, whereas as a result of the heat treatment of ginger, it decreased and amounted to 10.0%, 26.4%, 5.4% and 10.2% for each compound, respectively. In contrast, more camphene and α-curcumene were found in the processed ginger oil. After drying, the camphene content in the oil increased from 6.5% (the amount in the fresh ginger oil) to 7.6%, whereas the α-curcumene content increased from 5.8% to 6.0%. Interestingly, the content of geranial in both oils was at the same level and amounted to 6.6%.

Gümüşay and co-investigators studied the composition of ginger dried in a vacuum oven. Compared to the fresh ginger extract, the content of individual components in the dried ginger extract was 99.13%, 98.33% and 78.97% lower in the case of glutathione, cysteine and TPC, respectively. Additionally, the ascorbic acid concentration was 79.86 ± 4.37 mg/100 g DW, whereas in the dried ginger this component was not detected [55].

In the next work, the influence of the vacuum-drying of ginger on the composition of its essential oil constituents was investigated. In total, 54 compounds were identified in fresh ginger oil, whereas as many as 65 were identified in vacuum-dried ginger oil. Moreover, the quantitative changes in the main components of the oil occurred as a result of the treatment of ginger. The content of 2,6-octadienal,3,7-dimethyl-,(Z) and 2,6-octadienal, 3,7-dimethyl- in fresh ginger oil was 5.56 and 15.71%, respectively, whereas after drying it decreased down to 3.71 and 7.77%. On the other hand, the content of benzene,1-(1,5-dimethyl-4-hexenyl)-4-methyl- increased from 2.85 to 5.12%, 1,3-cyclohexadiene,5-(1,5-dimethyl-4-hexenyl)-2-methyl-[S-(R*,S*)] from 28.12 to 45.02%, α-farnesene from 6.90 to 11.50% and cyclohexene,3-(1.5-dimethyl-4-hexenyl)-6-methylene-,[S-(R*,S*)] from 7.64 to 11.32% [58].

#### 3.6.4. Drying in a Drying Chamber—Blanching as Pretreatment

Thuwapanichayanan et al. (2014) [62] investigated the effect of blanching, drying, and a combination of both of these heat treatments on the TPC content of ginger extract. The TPC in the fresh ginger extract was 15.41 ± 1.05 mg GAE/g DW, whereas as a result of blanching, this parameter decreased to 11.60 ± 1.18 mg GAE/g DW. Ginger samples dried only at 60, 70 and 80 °C, and samples first blanched and then dried at the given temperatures had a higher TPC than fresh ginger extract. The total phenolic content of the ginger extracts dried at each temperature was higher for the samples subjected to drying only compared to the samples subjected to pretreatment (blanching) and subsequent drying. The TPC value for ginger extract subjected to drying only was 18.71 ± 0.12 mg GAE/g (60 °C), 18.62 ± 0.04 mg GAE/g (70 °C) and 19.0 ± 0.17 mg GAE/g DW (80 °C), whereas for samples subjected to a two-stage thermal treatment, it was, for a given temperature, 16.90 ± 0.078, 17.76 ± 0.05 and 16.90 ± 0.63 mg GAE/g DW, respectively. These results show that drying has a positive effect on TPC, whereas the blanching process causes the loss of these compounds in ginger. In turn, the combination of both of these treatment methods gives intermediate results (Table 1).

The analysis of the scientific data shows that the short drying time of the rhizome at temperatures up to 60 °C has a positive effect on the determined amount of polyphenolic compounds. It seems interesting that the use of a variable temperature in the range of 30–40 °C with a variable humidity setting from 10% to 30% increases the amount of 6-gingerol. The drying time exceeding 36 h will, in most cases, decrease the amount of the determined compounds in the ginger rhizome. Based on the studies, it can be also noted that the choice of temperatures and drying time will significantly affect the quantity and quality of the essential oil. In most cases, a higher temperature allowed us to obtain more oil, but it strongly influenced its quality.

### 3.7. Heating in a Thermostatic Bath/Heating in the Oven/Heating

In addition to drying, in some studies the process of heating the ginger rhizomes was used, and this method’s influence on the content of individual ginger compounds in the rhizome was investigated. From the results of Yamaguchi et al. (2010) [63], it can be concluded that the heat treatment of ginger in a thermostated water bath at 100 °C for 10 min had a positive effect on the volatile component profile of ginger. After the heat treatment, an increase in the concentration of 22 aromatic compounds was observed, whereas the concentration of only 9 compounds decreased. The highest increase was noticed in the case of α-zingiberene of nearly 2.5 times (from 2.56 to 8.94 mg/kg). Significant increases were also noted for neral (5.17 to 12.85 mg/kg) and zingerone (3.55 to 8.54 mg/kg). In turn, the largest decrease in the content was recorded for *β*-bisabolene (214.42 to 126.75 mg/kg), α-cubebene (0.87 to 0.57 mg/kg) and citronellal (0.12 to 0.08 mg/kg) (Table 1). In turn, Schweiggert et al. (2005) [42] investigated if essential oil content decreased in ginger samples that were only ground or ground and then moderately heated compared to the fresh plant, which contained 1.20% DW. These values were 0.30% DW (fragmented sample), 0.59% DW (sample ground and heated at 80 °C for 10 min), 0.51 (±0.03)% DW (sample ground and heated at 90 °C for 5 min). Cell destruction and heating may be responsible for the loss of volatile oil, since volatile components are sensitive to temperature rise [64]. Interestingly, heating the ground ginger under different conditions resulted in a much higher retention of the essential oil. The content results were as follows: heating for 10 min at 90 °C—0.93% DW; for 1 min at 100 °C—1.19% DW; and for 10 min at 100 °C—1.25% DW [47]. Therefore, based on the presented results, it may be concluded that the influence of heating on the content of the ginger essential oil is inconclusive, depending on time and the temperature used.

In another article, Khatun et al. (2006) [64] revealed that the TPC of fresh ginger decreased in each case from 20.0 ± 0.5 to 15.0 ± 0.5 (µmol GAE/g) as an effect of heating the ginger in the oven for 1, 3 or 6 h.

To sum up, it can be concluded that according to the researchers, heating of the rhizome at 100 °C for 10 min provides an increased content of essential oils. However, these conditions induce qualitative changes to its composition. A longer heating (1–3 h) at the same temperature resulted in a decrease in the content of polyphenolic compounds.

### 3.8. Microwave Processing

During thermal processing with the use of microwaves, the generated heat is a result of the action of an alternating electric field on the product. This causes migration of ions and vibration of dipolar molecules, which results in the generation of heat in the product. Therefore, it is expected that this type of treatment will provide a more homogeneous heat in a shorter time compared to conventional methods such as, e.g., drying the product with hot air or drying in the sun [53].

In the study of Kubra and Rao (2011) [59], the microwave drying of ginger resulted in a reduction in the amount of essential oil compared to its content in the fresh rhizome (3.2% *v*/*w*). The content of oil in the microwave-dried rhizomes was, as follows, for a power level of (PL) 100 (800 W), 3.0% *v*/*w*; PL 80 (660 W), 2.3% *v*/*w*; PL 60 (525 W), 2.0% *v*/*w*; and PL 40 (385 W), 2.3% *v*/*w*. The content of monoterpene hydrocarbons also decreased from 619 µL/100 g DW for fresh ginger to 421 for PL 100, 366 for PL 80, 533 for PL 60 and 491 µL/100 g DW for PL 40. The concentration of oxygenated monoterpenes has also significantly changed from 405 µL/100 g DW (for the unprocessed rhizome) to 171, 182, 213 and 241 µL/100 g DW for the given parameters. It can be seen that in the case of these compounds, their content increased with the decrease in the power level of the microwaves used during drying. There was also a significant loss of other aliphatic compounds in the essential oil during the thermal treatment. Their content in the fresh rhizome was 6 µL/100 g DW, whereas with the decrease in the microwave radiation power, it reached the following values: 2, 2, 4 and 3 µL/100 g DM for 100, 80, 60 and 40% of the PL, respectively. In contrast, for other oxygenated aliphatic compounds, the situation was 37 µL/100 g DW for unprocessed rhizomes, followed by 28, 22, 23 and 25 µL/100 g DW, for the respective PLs. However, as a result of the thermal treatment, the content of oxygenated sesquiterpenes increased and was equal to 146 µL/100 g DW in fresh ginger oil, and with the decrease in microwave power, it reached the following values: 190, 163, 165 and 173 µL/100 g DW for the given PLs. The changes in the content of sesquiterpene hydrocarbons in the studied samples were also interesting. Microwave-dried ginger rhizome at PL 100 was the most rich in essential oil, where the concentration of these compounds was 2149 µL/100 g DW. The second most rich was essential oil from fresh ginger (1963 µL/100 g DW), followed by ginger processed at PL 80 (1534 µL/100 g DW), PL 40 (1330 µL/100 g DW) and PL 60 (1040 µL/100 g DW) (Table 1).

In another study, essential oil from microwave-dried ginger contained 48 compounds, whereas fresh ginger oil contained 49 compounds. Due to the thermal treatment, various types of changes in the content of individual components of the oils occurred. The concentration of some compounds increased, whereas others decreased. It can be noticed by comparing the content of selected substances which were classified as the main components of both oils. The concentration of zingiberene, geranial, *β*-sesquiphellandrene, α-curcumene and *β*-bisabolene in the fresh oil was as follows: 22.76, 14.50, 7.01, 2.78 and 3.25%, whereas in microwave-dried ginger oil, the concentration was 40.10, 4.96, 10.94, 6.21 and 5.64% for the mentioned compounds, respectively. As a result of the heat treatment, the content of gingerols decreased significantly. The concentration of 6-gingerol decreased from 5.91 mg/g DW in fresh ginger extract to 2.12 mg/g DW in dried ginger extract, 8-gingerol from 2.52 to 1.35 mg/g DW, and 10-gingerol from 2.62 to 1.05 mg/g DW. On the other hand, microwave drying contributed to a visible increase in the content of 6-shogaol from 0.09 to 0.384 mg/g DW. This type of treatment adversely affected the TPC and TFC. TPC decreased from 11.97 ± 0.33 mg GAE/g DW in the extract from fresh ginger to 8.41 ± 0.35 mg GAE/g DW in the processed plant. In turn, the TFC in these extracts was at the level of 13.49 ± 0.36 and 12.55 ± 0.74 mg rutin/g DW for fresh and dried rhizomes, respectively [57].

According to Lim and Murtijay (2007) [65], the strong thermal degradation of phenolic compounds during microwave drying could be caused by the rapid and intense heat generated during the drying process. In addition, the activation of oxidative enzymes (under the influence of drying) such as peroxidases and polyphenol oxidases may have contributed to the loss of phenol complexes.

In the studies of Ding et al. (2012) [58], it turned out that drying ginger in a microwave had a positive effect on the diversity of the volatiles of its essential oil. Fifty-four volatile compounds were identified in fresh ginger oil, whereas in the processed ginger oil, fifty-nine were. This type of treatment reduced the content of 2,6-octadienal, 3,7-dimethyl-,(Z) oil from 5.56% (in oil from fresh ginger) to 2.92% (in microwave-dried ginger oil) and 2,6-octadienal, 3,7-dimethyl- from 15.71 to 10.27%. On the other hand, as a result of drying, the content of the other main components of the oil increased, e.g., benzene,1-(1,5-dimethyl-4-hexenyl)-4-methyl- increased from 2.85 to 5.31%, 1.3-cyclohexadiene,5-(1,5-dimethyl-4-hexenyl)-2-methyl-[S-(R*,S*)] from 28.12 to 45.16%, α-farnesene from 6.90 to 11, 72% and cyclohexene,3-(1,5-dimethyl-4-hexenyl)-6-methylene-,[S-(R*,S*)] from 7.64 to 11.35%.

In the work of Huang et al. (2012) [66], the composition of the essential oil from fresh ginger and ginger dried in a microwave oven was compared. Using the headspace solid-phase microextraction (HS-SPME) coupled to the gas chromatography-mass spectrometry (GC-MS) method, 42 components were identified in fresh ginger oil, whereas only 37 were identified in the oil from a processed rhizome. Heat treatment also influenced the composition of the ginger essential oil. In fresh ginger oil, the content of camphene was 6.5%, *β*-phellandrene 12.9% and geranial 6.6%, whereas dried ginger oil was characterized by a lower content of these compounds, which was: 2.5%, 7.4% and 0.9% for each compound, respectively. On the other hand, as a result of the thermal treatment the content of other terpenes increased—α-curcumene from 5.8% to 8.5%, zingiberene from 27.8% to 37.1%, *β*-bisabolene from 5.7% to 12.8% and *β*-sesquiphellandrene from 10.4% to 12.8%.

Most studies using microwave drying have shown a negative effect of high power levels and drying times on the content of ginger compounds. The longest presented process lasted 320 min at the power of 60 W and caused a reduction in the content of mostly volatile compounds and a change in their composition. The use of an energy density of 5 w/g in microwave drying resulted in a significant decrease in the content of polyphenolic compounds and ginger volatile compounds.

#### Intermittent Microwave-Convection Drying

Intermittent microwave-convection drying is a drying method that combines the action of warm air and microwaves to be used on plant material. In the study of An et al. (2016) [57], in the essential oil of fresh ginger, 49 volatile compounds were identified, whereas in the oil obtained from ginger subjected to intermittent microwave-convection drying, only 44 were found. As a result of the treatment, quantitative changes in individual components of the oil were also revealed. It was observed that the processing can cause both an increase and a decrease in the content of certain substances. The content of a few selected compounds among the main components of the oil was as follows: zingiberene, 22.76 and 42.20%; geranial, 14.50 and 7.31%; *β*-sesquiphellandrene, 7.01 and 11.24%; α-curcumene, 2.78 and 5.82%; and *β*-bisabolene, 3.25 and 5.93%, in fresh and intermittent microwave-convection dried ginger, respectively. Moreover, this drying method reduced the concentration of 6-, 8- and 10-gingerol in the ginger extract from 5.91, 2.52 and 2.62 mg/g DW to 3.21, 2.43 and 2.5 mg/g DW for the respective compounds. In turn, as a result of this type of thermal treatment, the content of 6-shoagol in the extract increased from 0.09 to 0.243 mg/g DW. Due to drying, the TPC parameter insignificantly decreased from 11.97 ± 0.33 to11.28 ± 0.40 mg GAE/g DW. It is interesting that intermittent microwave-convection drying positively influenced the total content of flavonoids (TFC) in the extract, increasing their content from 13.49 ± 0.36 to 15.42 ± 0.87 mg rutin/g DW for fresh and processed ginger rhizomes (in processed ginger extract) (Table 1).

### 3.9. Carbonization

Carbonization is the process of converting organic matter, such as food, into carbon during heating or through a chemical treatment. Li et al. (2016) [52] noticed that extract obtained from ginger after carbonization contained higher amounts of 6-gingerdiol, 6- and 8-paradol, 8- and 10-shogaol, 5-hydroxy-1-(4-dihydroxy-3-methoxyphenyl)-7-(3,4-dihydroxyphenyl)-3-heptanone and 3- or 5-acetoxy-6-gingerdiol in comparison to fresh ginger extract. Moreover, the heat treatment also contributed to an increase in the extract’s content of the five main constituents of ginger. The content of zingerone increased from the level below the LOQ (limit of quantification) to 2.745 ± 0.007 mg/g, 6-gingerol from 2.035 ± 0.023 to 3.049 ± 0.027 mg/g, 8-gingerol from 0.128 ± 0.010 to 0.840 ± 0.011 mg/g, 6-shogaol from 0.067 ± 0.005 to 7.658 ± 0.015 mg/g and 10-gingerol from 0.058 ± 0.002 to 1.143 ± 0.011 mg/g. After carbonization, the TPC parameter also increased by almost twice as much, from 8.46 to 16.37 mg GAE/g, in comparison to the extract from the fresh plant (Table 1).

**Table 1:** Effect of traditional processing on the content of secondary metabolites in ginger.

Lp	Type of Processing	Fresh Rhizome Processing Conditions	Tested Compounds	Changes in the Content	References
1	Roasting	13 min, 200 °C, Teflon-coated pan followed by freezing in liquid nitrogen	Volatile components	↓ (S)-linalool, (E)-2-octenal ↑ (S)-citronellal, geraniol, 3-hydroxy-4,5-dimethyl-2(5H)-furanone, 4-hydroxy-2,5-dimethyl 3(2H)-furanone, vanillin, 3-(methylthio)propanal, neral, (E)-2-octanal	[39,40]
2	Blanching	Air-drying at 25± 2 °C (1–2 h), peeling, slicing and blanching in boiling water with 2% citric acid (10 min), followed by 60 min drying at 50 °C	Total phenolic content (TPC)	↑ total phenolic content	[41]
3		10 min, 100 °C, blanching, followed by freeze-drying for 1.5 h at −30 °C in 100 mbar, then for 8.5 h at 20 °C in 45 mbar, for 5 h at 35 °C in 35 mbar, and 35 °C in 15 mbar for 55 h	Essential oil content	↑ essential oil content in processed rhizome	[32]
4		5, 15 and 30 min, 70 °C blanching and drying at 40 °C (rel. humidity 9–12%)	6-gingerol	↓ 6-gingerol	[43]
5	Steam cooking	120 min, 97 °C, 3 kgf/cm^3^ steaming and oven drying for 40 h at 45 °C	1-dehydro-6-gingerdione	↑ 1-dehydro-6-gingerdione	[44]
6		18 h steaming and freeze-drying for 7 days at −70 °C; microwave extraction	Total soluble solid yield (TSSY), 6-gingerol and 6-shogaol	↓ TSSY and 6-gingerol ↑6-shogaol	[45]
7	Steam heating	2 h steam heating and drying	Essential oil components	↓ citral, 1,8-cineole, sabinene ↑ *β*-sesquiphellandrene, farnesene, zingiberene, *ar-*curcumene, *β*-bisabolene, geranyl acetate, camphene	[49]
8		2 h steam heating and drying; ethyl acetate extraction	Volatile components	↓ camphene, citral, 1,8-cineole, sabinene ↑ *β*-sesquiphellandrene, farnesene, zingiberene, *ar-*curcumene, *β*-bisabolene, geranyl acetate	[49]
9	Stir-frying	Dried rhizome (40 °C), stir-frying for 7 min at 220 °C; methanol extraction	Phenolic components	↑ zingerone, 6-gingerol, 8-gingerol, 6-shogaol, 10-gingerol	[52]
10	Air-drying	1-week-long drying	Essential oil components	↓ majority of volatiles in the essential oil ↑ citral	[49]
11		Air-drying in the sun	Phenolic compounds	↑ sum of phenolic compounds ((+)-catechin + (−)-epicatechin + rutin + myricetin + trans-resveratrol + quercetin + naringenin + kaempferol) ↑ TPC	[54]
12		3-day-long air-drying in the sun (at 25–30 °C)	Glutathione, cysteine, ascorbic acid and TPC	↓ glutathione, cysteine, ascorbic acid and TPC	[55]
13	Drying	Drying at 30–60 °C in relative humidity of 10–30%	6-gingerol	↓ 6-gingerol Increase in temperature and stable drying conditions decreased its content faster	[43]
14		Dried ginger powder	Phenolic components	↓ 6-, 8-, 10-gingerol ↑ 6-shogaol	[56]
15		Drying at 40 °C, followed by methanol extraction	Phenolic components	↑ zingerone, 6-, 8-, 10-gingerol, 6-shogaol	[52]
16	Hot-air-drying	Drying at 60 °C in an electric thermostatic oven	Essential oil, phenolic components	↓ 6-, 8-, 10-gingerol, TPC, TFC (total flavonoid content) ↑6-shogaol	[57]
17		Drying at 50, 60 or 70 °C in an electric-heating air blast dryer (air flow velocity of 0.75 ± 0.03 m/s)	Essential oil components	↓ 2,6-octadienal, 3,7-dimethyl-, (Z), 2,6-octadienal, 3,7-dimethyl- ↑ benzene, 1-(1,5-dimethyl-4-hexenyl)-4-methyl-, 1,3-cyclohexadiene, 5-(1,5-dimethyl-4-hexenyl)-2-methyl-[S-(R*,S*)], α-farnesene, cyclohexene, 3-(1,5-dimethyl-4-hexenyl)-6-methylene-,[S-(R*,S*)]	[58]
18	Convection drying	12 h, 50 ± 4 °C with hourly agitation	Volatile components	↓ zingiberene, γ-muurolene, α-farnesene, *β*-sesquiphellandrene ↑ *β*-phellandrene, camphene, neral, geranial, *ar*-curcumene	[59]
19	Oven drying	Drying at 40, 50, 60 and 70 °C	Phenolic compounds	↑ sum of phenolic compounds ((+)-catechin + (−)-epicatechin + rutin + myricetin + trans-resveratrol + quercetin + naringenin + kaempferol) ↑ TPC	[54]
20		36 h, 60 °C	Glutathione, cysteine, ascorbic acid and TPC	↓ glutathione, cysteine, ascorbic acid and TPC	[55]
21		36 h, 60 °C, then 48 h at 80 °C	Ginger components	↓ 6-, 8-, 10-gingerol, methyl-6-gingerol, methyl-8-gingerol, methyl-10-gingerol, 5-acetocy-6-gingerdiol, acetoxy-6-gingerol, diacetoxy-6-gingerdiol, gingerenone A and B, hexahydrocurcumin, 6-gingerdiol 5-glucopyranoside, 6- and 8-paradol, methyl-8-paradol ↑ phenylalanine, cinnamic acid, 6-shogaol, =6-gingerdiol	[60]
22		1 h, 80 °C in an oven (1000 W)	Essential oil components	↓*β*-phellandrene, zingiberene, *β*-bisabolene, *β*-sesquiphellandrene ↑camphene, *α*-curcumene =geranial	[66]
23	Vacuum-drying oven	36 h, 60 °C at 0.025 mbar	Glutathione, cysteine, ascorbic acid and TPC	↓ glutathione, cysteine, ascorbic acid and TPC	[55]
24		490 min, 60 °C, at 13.3 kPa	Essential oil components	↓ 2,6-octadienal, 3,7-dimethyl-, (Z), 2,6-octadienal, 3,7-dimethyl- ↑ benzene, 1-(1,5-dimethyl-4-hexenyl)-4-methyl-, 1,3-cyclohexadiene, 5-(1,5-dimethyl-4-hexenyl)-2-methyl-[S-(R*, S*)], *α*-farnesene, cyclohexene, 3-(1,5-dimethyl-4-hexenyl)-6-methylene-,[S-(R*,S*)]	[58]
25	Blanching as pretreatment and drying in a heater	Slices blanched 2 min at 95 ± 1 °C and dried at 60, 70 or 80 °C at a superficial air velocity of 0.3 m/s in the drying chamber	TPC	↓ TPC	[62]
26	Heating	10 min, 100 °C water bath, followed by ice-water-bath cooling	Volatile components in diethyl ether extract	↑ most of the volatile components ↓ citronellal, *β*-bisabolene, *β*-sesquiphellandrene, *α*-farnesene	[63]
27		Heating in a hermetically sealed 3 L pilot-scale reaction vessel at 80 °C for 10 min, 90 °C for 5 and 10 min, and 100 °C for 1 and 10 min, followed by freeze-drying for 1.5 h at −30 °C in 100 mbar, then for 8.5 h at 20 °C in 45 mbar, for 5 h at 35 °C in 35 mbar, and 35 °C in 15 mbar for 55 h	Essential oil components	The essential oil content changed depending on the heating conditions	[42]
28		1, 3 or 6 h heating at 100 °C in the oven (1 g of ginger in 20% ethanol)	TPC	↓ phenolic content	[64]
29	Microwave drying	25–48 min, 2450 MHz (230 V, 50 Hz, 1350 W ± 10%)	Volatile components	↓ monoterpenes, sesquiterpenes ↑eudesmol, cubenol and cedrene derivatives, *ar*-curcumene, epiglobulol, nerolidol	[59]
30		Energy density of 5 W/g until moisture content reached 1.0 g H_2_O/g DW, then with 1 W/g to the terminal point	Essential oil and phenolics	↓ phenolics and flavonoids ↓volatiles ↑ 6-shogaol, cycloisosativene, copaene, *gamma*-elemene	[57]
31		320 min, 2455 MHz, 60 W	Volatile components	↓ volatiles, (2,6-octadienal, 3,7-dimethyl-, (Z), 2,6-octadienal, 3,7-dimethyl-) ↑benzene, 1-(1,5-dimethyl-4-hexenyl)-4-methyl-, 1,3-cyclohexadiene, 5-(1,5-dimethyl-4-hexenyl)-2-methyl-[S-(R*,S*)],*α*-farnesene, cyclohexene, 3-(1,5-dimethyl-4-hexenyl)-6-methylene-,[S-(R*,S*)]	[58]
32		2 min, 220 V, 50 Hz, 700 W	Essential oil components	↓ camphene, *β*-phellandrene, geranial ↑ *α*-curcumene, zingiberene, *β*-bisabolene, *β*-sesquiphellandrene	[66]
33	Intermittent microwave-convection drying	Drying at 60 °C in a laboratory 700 W microwave oven, 5 s on, 5 s off to 50% water content, then in 5 s on, 25 s off mode; supported by hot-air adjustment of the microwave pulse rate	Essential oil and phenolic content	↓ 6-, 8-gingerol ↑ 6-shogaol, TFC =10-gingerol, TPC	[57]
34	Carbonization	Drying at 40 °C, followed by heating and frying; extraction with methanol	Ginger components	↑ zingerone, 6-gingerol, 8-gingerol, 6-shogaol, 10-gingerol	[52]

RH—relative humidity. ↑—increased content; ↓—decreased content; =no effect on the content.

### 3.10. Other

#### 3.10.1. Lyophilization (Freeze-Drying)

Lyophilization or freeze-drying is a method of removing water from plant tissues using low temperatures. The process can also be carried out with a vacuum, i.e., under anaerobic conditions. Despite that it is a relatively expensive and time-consuming processing method, many producers choose this type of drying due to the high quality of the final product. Compared to other drying methods, freeze-drying allows the raw material to retain more biologically active compounds after drying [2].

The freeze-drying of 9- and 12-month-old ginger rhizomes positively influenced the content of the sum of phenolic compounds ((+)-catechin, (−)-epicatechin, rutin, myricetin, trans-resveratrol, quercetin, naringenin and kaempferol) in their extracts. In the 9-month-old fresh ginger, the concentration of the above-listed components was 38.43 mg/100 g, whereas after lyophilization the concentration significantly increased to 386.24 mg/100 g. Additionally, in the case of 12-month-old plants, the content increased from 55.32 to 379.08 mg/100 g. The situation was similar in the case of TPC measurements, which increased from 21.84 ± 3.21 to 174.30 ± 1.32 mg GAE/g and from 39.81 ± 2.25 to 179.29 ± 3.32 mg GAE/g for 9- and 12-month-old rhizomes, respectively (Table 2) [54].

The increase in the phenols’ content after lyophilization may be caused by the formation of ice crystals during freezing, which may lead to a greater disturbance of the cell wall structure. Damage to the cell wall, in turn, promotes the penetration of the solvent into the cell during the extraction and accelerates the release of cellular components, including secondary metabolites [67].

On the other hand, Gümüşay et al. (2015) [55] reported that the freeze-drying of ginger contributed to the loss of some components in the extract, in comparison to the fresh ginger extract. Glutathione content was reduced by 72.58%, cysteine by 32.21% and TPC by 32.58%. The fresh ginger extract also contained ascorbic acid in the amount of 79.86 ± 4.37 mg/100 g DM, which was completely lost as a result of freeze-drying.

The freeze-drying of ginger also affects the composition of its volatile substances. In the studies by An et al. (2016) [57], it was found that 49 of its volatile compounds were detected during the qualitative analysis of fresh ginger oil, and only 41 were present in the oil after lyophilization. The content of some main components in both oils (zingiberene, geranial, *β*-sesquiphellandrene, α-curcumene and *β*-bisabolene) was as follows: 22.76, 14.50, 7.01, 2.78 and 3.25% and 41.64, 6.65, 10.79, 5.79 and 5.77% in oil from fresh and lyophilized ginger rhizomes, respectively. A thorough analysis revealed that the treatment resulted not only in some qualitative changes, but also quantitative changes. Due to the chemical nature of individual components, freeze-drying contributed to an increase or decrease in their concentration. Fresh ginger extract contained more 6-gingerol (5.91 mg/g DW) than the processed ginger extract (3.54 mg/g DW). Other changes were insignificant. The concentration of 8-gingerol did not change and the content of 10-gingerol increased from 2.62 to 2.72 mg/g DW for fresh and lyophilized ginger, respectively. It was expected that the processing of ginger would contribute to an increase in the amount of 6-shogaol in the extract. Its concentration after treatment increased to 0.221 mg/g DW, in comparison to 0.09 mg/g DW in the fresh rhizome. This type of processing had also a positive effect on the TPC parameter of the extract. Freeze-drying increased the TPC from 11.97 ± 0.33 up to 13.83 ± 0.31 mg GAE/g DW. On the other hand, this process did not affect the TFC parameter, as the values were almost the same for fresh and processed ginger, such as 13.49 ± 0.36 and 13.32 ± 0.52 mg rutin/g DW, respectively.

In the studies of Ding et al. (2012) [58], the exposure of ginger to cold temperatures caused qualitative and quantitative changes in its essential oil. In freeze-dried ginger oil, 50 volatile components were identified, compared to 54 compounds assigned in fresh ginger oil. The content of two compounds, 2,6-octadienal, 3,7-dimethyl-,(Z) and 2,6-octadienal, 3,7-dimethyl-, decreased from 5.56 to 2.27% for the former and from 15.71 to 15.11% for the latter. On the other hand, the concentration of benzene,1-(1,5-dimethyl-4-hexenyl)-4-methyl- increased from 2.85 to 5.40% in the freeze-dried ginger oil compared to the fresh ginger oil; 1,3-cyclohexadiene,5-(1,5-dimethyl-4-hexenyl)-2-methyl-[S-(R*,S*)] from 28.12 to 41.46%; α-farnesene from 6.90 to 12.02%; and cyclohexene,3-(1,5-dimethyl-4-hexenyl)-6-methylene-,[S-(R*,S*)] from 7.64 to 11.54%.

Freeze-drying conducted at −40 °C induced an increase in polyphenolic component content; however, lower temperatures of the process, such as −50 or −58 °C, led to a decrease in the content of the studied metabolites.

#### 3.10.2. Infrared Drying

Infrared (IR) drying has recently become an alternative food-drying technique. In this method, infrared radiation penetrates the matter, turning into heat and removing water. This type of drying can be used as a single technique or in addition to other drying methods. It has many advantages, such as energy efficiency and the high quality of the obtained products [68,69].

In the study of An et al., fresh ginger essential oil was found to contain 49 compounds in comparison with the infrared-dried ginger oil that had 47 identified components. Apart from qualitative differences, quantitative analyses revealed marked differences between the tested samples. The content of selected compounds was as follows: zingiberene, 22.76 without and 38.81% with processing; geranial, 14.50 and 11.73%; *β*-sesquiphellandrene, 7.01 and 10.02%; α-curcumene, 2.78 and 5.54%; and *β*-bisabolene, 3.25 and 5.30%, respectively. The obtained results revealed that IR processing contributed to various changes in the content, particularly of volatile compounds; however, some changes in the phenolic content were also observed. The quantity of 6-gingerol decreased from 5.91 to 3.44 mg/g DW, 8-gingerol from 2.52 to 2.48 mg/g DW and 10-gingerol from 2.62 to 2.52 mg/g DW. On the other hand, similar to other studies, the content of 6-shogaol increased from 0.09 to 0.209 mg/g DW. As a result of drying, the TPC decreased from 11.97 ± 0.33 to 11.35 ± 0.66 mg GAE/g DW. However, the TFC parameter was higher for the processed rhizome in comparison to the fresh plant (14.52 ± 0.23 and 13.49 ± 0.36 mg rutin/g DW for processed and fresh ginger, respectively) (Table 2) [57].

The presented review revealed that, depending on the drying method, various changes in the composition and concentration of volatile substances were observed. However, regardless of the type of heat treatment, in a majority of the studies it was revealed that the content of monoterpenes decreased and the concentration of sesquiterpenes increased upon the processing. Probably, these changes in the oil composition were caused by the synthesis of short-chain alkenes and the isomerization of similar compounds [57].

By analyzing the data from the presented publications, it can be seen in most cases that more compounds were identified in oils obtained from processed rhizomes than in oils from fresh raw material, which may be due to the formation of different esters, e.g., as a result of the esterification of alcohols. The formation of new compounds and the disappearance of compounds originally present in the raw material were also noticed, as a result of chemical rearrangements under the influence of oxidation processes that take place at an elevated temperature. In addition, a prolonged exposure to high temperatures may contribute to the breakdown of sesquiterpene compounds into monoterpenes [57,58]. Most studies noted that in the oils obtained after the processing of the rhizome, higher concentrations were found for *ar*-curcumene, *β*-bisabolene, *β*-sesquiphellandrene and zingiberene, whereas the content of geranial decreased.

For most drying methods (hot-air, infrared, microwave, freeze-drying, and microwave-convection drying, with the exception of microwave drying), 6-gingerol was more labile than 8- and 10-gingerol, which resulted in a significant decrease in its content compared to other gingerols [57]. It was proved that temperature is the most important factor in thermochemical conversion processes [70]. As a result of thermal treatment, the *β*-hydroxy-ketone groups of gingerol become dehydrated and the corresponding shogaol derivatives are formed. Additionally, *β*-unsaturated ketones in shogaols are thermodynamically stable, and thus, these compounds are much less susceptible to changes caused by heat [71]. However, the exposure of shogaols to excessively high temperatures (e.g., 180 °C) leads to a decrease in their content. This phenomenon could be a result of a long-term heating process that resulted in the degradation and polymerization of shogaols [16]. Additionally, at high temperatures, water evaporates faster, and as studied, the transformation of gingerols into shogaols takes place in the aqueous environment; therefore, the transformation of these compounds may be inhibited due to a water loss that is too intensive [72,73]. It is also interesting that the transformation of gingerols into shogaols is catalyzed by acids, which in water solutions create various types of anions that accelerate this process [16,74].

In one of the studies, the stability of 6-gingerol and 6-shogaol in aqueous solutions at pH 1 was assessed under the influence of temperature changes ranging from 37 to 100 °C. It has been noticed that under the influence of temperature, 6-gingerol may be dehydrated into 6-shogaol, but this compound may be again hydrated and converted back to 6-gingerol. It was confirmed that the degradation of 6-gingerol happens according to a reversible kinetics. According to this study, 6-gingerol was relatively stable up to 37 °C, whereas at temperatures above 60 °C the degradation degree was 50%. In turn, 6-shoagol is more resistant to high temperatures and the loss of this compound at the level of 50% occurs at a temperature of 80 °C [47].

## 4. Conclusions and Perspectives

Based on the collected results from the presented publications on the effect of thermal treatments on the chemical composition of ginger rhizomes, it can be stated that the methods and conditions of treatment have a significant impact on the qualitative and quantitative composition of the obtained extract, both on its volatile and polyphenolic fractions. Interesting technologies are freeze-drying and infrared heating, but they require a higher expenditure of energy and drying time, and more expensive equipment. Undoubtedly, the selection of the appropriate processing method affects the quality of the product and the efficiency of the process and, thus, there is a wide variety of applications in the food and medical industry.

## Figures and Tables

**Table 2:** Effect of lyophilization and infrared drying (unconventional methods) on the content of secondary metabolites in ginger.

Lp	Type of Processing	Processing Conditions	Tested Compounds	Changes in the Content of Compounds	Reference
1	Freeze-drying/lyophilization	Freeze-drying at −40 °C	Phenolic compounds	↑ sum of ((+)-catechin + (−)-epicatechin + rutin + myricetin + trans-resveratrol + quercetin + naringenin + kaempferol) ↑ TPC	[54]
2		Freeze-drying for 24 h at −50 °C under a pressure of 0.133 mbar	Content of glutathione, cysteine, ascorbic acid and TPC	↓ glutathione, cysteine, ascorbic acid and TPC	[55]
3		Freezing for 12 h at −40 °C and freeze-drying at 20 Pa using 25 and −58 °C for a heating plate and cold trap	Phenolic components	↓ 6-gingerol = 8- and 10-gingerol, TFC ↑ 6-shogaol, TPC	[57]
4		4 h at −80 °C, followed by vacuum-drying for 29 h at 0.203 kPa, at temp. of 22 °C and −55 °C	Essential oil components	↓ (2,6-octadienal, 3,7-dimethyl-, (Z), 2,6-octadienal, 3,7-dimethyl-) or increased (benzene, 1-(1,5-dimethyl-4-hexenyl)-4-methyl-, 1,3-cyclohexadiene, 5-(1,5-dimethyl-4-hexenyl)-2-methyl-[S-(R*,S*)], α-farnesene, cyclohexene, 3-(1,5-dimethyl-4-hexenyl)-6-methylene-, [S-(R*,S*)])	[58]
5	Infrared drying	Drying with three red glass lamps (225 W each)	Phenolic components	↓ 6-, 8-, 10-gingerol, TPC ↑ 6-shogaol, TFC	[57]

↑—increased content; ↓—decreased content; =no effect on the content; temp.—temperature.

## Data Availability

Not applicable.
